# A 6-day high-intensity interval microcycle improves indicators of endurance performance in elite cross-country skiers

**DOI:** 10.3389/fspor.2022.948127

**Published:** 2022-11-09

**Authors:** Bent R. Rønnestad, Kjetil Andre Bjerkrheim, Joar Hansen, Knut Sindre Mølmen

**Affiliations:** Section for Health and Exercise Physiology, Inland Norway University of Applied Sciences, Lillehammer, Norway

**Keywords:** endurance training, high-intensity aerobic training, intense exercise, high-intensity intermittent training, roller skiing

## Abstract

**Purpose:**

The aim of this study was to compare the effects of a 6-day high-intensity interval (HIT) block [BLOCK, *n* = 12, maximal oxygen uptake (V̇O_2max_ = 69. 6 ± 4.3 mL·min^−1^·kg^−1^)] with a time-matched period with usual training (CON, *n* = 12, V̇O_2max_ = 69.2 ± 4.2 mL·min^−1^·kg^−1^) in well-trained cross-country (XC) skiers on physiological determinants and indicators of endurance performance. Furthermore, the study aimed to investigate the acute physiological responses, including time ≥90% of V̇O_2max_, and its associated reliability during repeated HIT sessions in the HIT microcycle.

**Methods:**

Before the 6-day HIT block and following 5 days of recovery after the HIT block, both groups were tested on indicators of endurance performance. To quantify time ≥90% of V̇O_2max_ during interval sessions in the HIT block, V̇O_2_ measurements were performed on the 1st, 2nd, and last HIT session in BLOCK.

**Results:**

BLOCK had a larger improvement than CON in maximal 1-min velocity achieved during the V̇O_2max_ test (3.1 ± 3.1% vs. 1.2 ± 1.6%, respectively; *p* = 0.010) and velocity corresponding to 4 mmol·L^−1^ blood lactate (3.2 ± 2.9% vs. 0.6 ± 2.1%, respectively; *p* = 0.024). During submaximal exercise, BLOCK displayed a larger reduction in respiratory exchange ratio, blood lactate concentration, heart rate, and rate of perceived exertion (*p* < 0.05) and a tendency towards less energy expenditure compared to CON (*p* = 0.073). The ICC of time ≥90% V̇O_2max_ in the present study was 0.57, which indicates moderate reliability.

**Conclusions:**

In well-trained XC skiers, BLOCK induced superior changes in indicators of endurance performance compared with CON, while time ≥90% of V̇O_2max_ during the HIT sessions in the 6-day block had a moderate reliability.

## Introduction

The importance of high-intensity interval training (HIT) for improving endurance performance in well-trained endurance athletes is established [e.g., (1)]. During the last decade, focus has been shed on the potential endurance performance benefits of block periodization (2) and in that regard has especially the effects of shorter periods with focus on specific training modalities, such as HIT, in well-trained endurance athletes been studied [e.g., (3)]. Specifically, a HIT microcycle has been observed to improve endurance performance-related variables in a within-group design (4–6) when the HIT microcycle contains a larger HIT volume than the control group (7–9) and when compared to a group that matches the overall HIT volume (10, 11). Despite that the superiority of HIT microcycles is not universal (12), one of the main arguments for using HIT and HIT microcycles is to improve maximal oxygen uptake (V̇O_2max_). For improving this variable, haematological variables are amongst the decisive factors (13), where a HIT session indeed is known to induce a transient increase in plasma volume (PV) (14). Moreover, it has been observed that three 1-week HIT microcycles can induce a moderate effect on haemoglobin mass (Hb_mass_) (3), but the effects of a single HIT microcycle on haematological variables have not yet been investigated in well-trained cross-country (XC) skiers. HIT is anecdotally termed as “V̇O_2max_ training” or “V̇O_2max_ intervals”. However, several studies investigating changes in V̇O_2max_ after a HIT microcycle report no significant changes compared to control conditions (6, 8, 10), while others did indeed observe a larger improvement in V̇O_2max_ (7, 9, 11). Interestingly, all cited studies did still find improvements in endurance performance or performance-related variables, indicating that for endurance-trained persons, a HIT microcycle may induce improvements in performance despite less pronounced or no change in V̇O_2max_.

Improved performance after a HIT microcycle without a concomitant change in V̇O_2max_ might be especially relevant for cross-country (XC) skiers, known to have amongst the highest V̇O_2max_ values ever recorded (15), thus making this variable difficult to improve (16). Accordingly, a study on elite XC skiers showed no change in V̇O_2max_ during an entire training year (17). Therefore, it is not certain that simply increasing the number of HIT sessions for only one training week, at the cost of total training hours and amount of low-intensity training (LIT), will improve performance. However, in a training volume and intensity matched study, we observed that XC skiers initiating a 5-week training period with a HIT microcycle achieved larger gains in endurance performance variables compared to the control group, despite no group differences in V̇O_2max_ changes (10). Of note, it has been suggested that HIT microcycles in a real-life context are utilised in order to increase the total HIT stimulus (18), but to the best of our knowledge, the effects of such a scenario in XC skiers remain to be investigated against a control group continuing their usual training. A case report on the Norwegian female national team in XC skiing described their block periodization of HIT across three continuous years (19). Unfortunately, no data on physiological adaptations were presented in that paper, but the International Ski Federation's ranking points showed improved performance during the 1st year which was maintained during the following years. In the latter study, a comprehensive amount of heart rate (HR) and blood lactate concentration [La^−^] data from the HIT blocks showed that the mean values during the 5 × 4 min work intervals were 91% of HR_max_ and 7.3 mmol·L^−1^, respectively (19). However, in that study and the literature in general, there is lacking measurements of V̇O_2_ during the HIT sessions in a microcycle. There seems to be growing evidence for the importance of accumulated time ≥90% of V̇O_2max_ to maximally stress cardiorespiratory parameters in well-trained endurance athletes (20–22), which also is an intensity domain known to induce a large muscular stimulus eventually leading to favourable muscular adaptations such as capillarization and improvements in mitochondrial functions (23, 24). However, there is a lack of knowledge on the reliability of accumulated time ≥90% of V̇O_2max_ between interval sessions, thus making it difficult to know whether the result for a training session tested in the laboratory will be similar for the next training sessions, or when the session will be repeated outside the laboratory during more practical everyday training. One study investigated this in runners and observed a relatively low absolute reproducibility (25), but to the best of our knowledge, the reproducibility of accumulated time ≥90% V̇O_2max_ from interval session to interval session has not previously been investigated for the movement pattern of roller ski skating.

Therefore, the primary purpose of this study was to compare the effects of performing a HIT microcycle vs. a time-matched period with usual training on physiological determinants of endurance performance in well-trained XC skiers. The secondary purpose was to investigate the acute physiological responses, including accumulated time ≥90% V̇O_2max_, to repeated HIT interval sessions during the HIT microcycle. Based on the limited literature specific to XC skiers, we hypothesised that (1) a HIT microcycle would improve performance measurement but not V̇O_2max_ and (2) the time accumulated ≥90% V̇O_2max_ during repeated HIT interval sessions in the HIT microcycle are distinguished by moderate reliability.

## Methods

### Participants

Twenty-nine well-trained male XC skiers volunteered for this study which was performed according to the ethical standards established by the Helsinki Declaration of 1975 and approved by the Local Ethical Committee of the Section for Health and Exercise Physiology, Inland Norway University of Applied Sciences (MR31072018). All participants provided informed consent. Three athletes dropped out throughout the intervention period due to illness, two withdrew due to personal reasons, and their data were subsequently excluded from the analysis. Thus, 24 athletes completed the study. A covariate adaptive randomisation was performed based on the covariates V̇O_2max_ and age to construct the two groups: BLOCK [*n* = 12; age, 21.2 (2.9) years; body mass, 75.1 (4.1) kg; body height, 182 (5) cm; V̇O_2max_, 69.6 (4.3) mL·min^−1^·kg^−1^] and CON [*n* = 12, age, 21.8 (2.2) years; body mass, 76.7 (5.7) kg; body height, 182 (5) cm; V̇O_2max_, 69.2 (4.2) mL·min^−1^·kg^−1^].

The study was performed in the month of August, i.e., ~3 months into the preparatory period for XC skiers and ~3 months before the start of the competition season. It was no group difference in mean training hours during the last month preceding the study. The distribution of endurance training time, categorised into a three-zone model [according to Sylta et al. (26)], was similar in BLOCK and CON for heart rate zone 1 [60–82% of maximal heart rate (HR_max_): 36.0 ± 18.8 vs. 29.3 ± 18.3 h, zone 2 (83–87% of HR_max_): 1.8 ± 1.9 vs. 1.4 ± 1.0 h, and zone 3 (88–100% of HR_max_): 1.7±1.2 vs. 2.0±1.6 h]. Power, plyometric, and heavy strength training constituted 5.9 ± 3.3 vs. 4.5 ± 3.0 h in BLOCK and CON, respectively.

### Experimental design

The main objective of the present study was to compare the effect of a short, 6-day HIT block with a time-matched period with usual training (CON) on indicators of endurance performance in well-trained XC skiers. The 6-day HIT block was composed of 5 HIT sessions which were followed by 5 days of recovery. For CON, they continued their usual training with the exception of the two last days preceding testing which was similar in BLOCK and CON. During the 6-day HIT block, the same HIT session with varied work intensity [as described in detail in Rønnestad et al. (27)] was used in all sessions, starting with one daily HIT session during the first three consecutive days, followed by one recovery day and finally 2 days in a row with one daily HIT session. To quantify accumulated time ≥90% V̇O_2max_ during the HIT block, V̇O_2_ during the intervals was measured on the 1st, 2nd, and 5th HIT sessions.

### Exercise testing protocol

The exercise testing protocol consisted of a blood lactate profile test followed by a V̇O_2max_ test, both performed using the XC skate skiing technique on roller skis. Despite all participants having previous experience with roller ski skating on treadmill, a familiarisation session was performed where the entire test protocol was conducted 2 days prior to the pretest. Pre- and posttesting for the individual XC skier was performed at the same time of day (±1 h) to avoid the influence of circadian rhythm. Roller ski testing and all HIT sessions (including the warm-up) were performed while skating on a treadmill (Rodby, RL3500E, Rodby Innovation. Uppsala, Sweden) using the same roller skis (IDT Skate Elite RM2, IDT Solutions AS, Lena, Norway), the same poles (SWIX Triac 2.0, Swix Sport AS, Lillehammer, Norway), and under similar environmental conditions (17–19°C) with a fan ensuring circulating air. The athletes used their ski boots and could freely choose between the two predominant uphill skating techniques (V1, where the skiers use their poles on every second leg push-off and V2, where the poles are used on every leg push-off).

After a 10-min warm-up on the treadmill at an inclination of 3% and a velocity of 12 km·h^−1^ the test started. A constant inclination of 7% was used during the test. The first 5-min bout started at a velocity of 10 km·h^−1^ and increased by 1 km·h^−1^ for each 5-min bout until a [La^−^] above 4.0 mmol·L^−1^ was measured. Capillary blood samples were taken from a fingertip during a 1-min break in between each 5-min bout and analysed for whole blood [La^−^] (Biosen C-line, EKF Diagnostics, Barleben, Germany). The average V̇O_2_ from the two last minutes of each 5-min bout was used for a subsequent calculation of maximal aerobic speed (MAS) based on the relationship between V̇O_2_ and workload. Energy expenditure during 10, 11, and 12 km·h^−1^ was calculated using energy equivalents from Péronnet and Massicotte (28). V̇O_2_ was measured with a sampling time of 30 s, using a computerised metabolic system with mixing chamber (Oxycon Pro, Erich Jaeger, Hoechberg, Germany). The flow turbine (Triple V, Erich Jeger) was calibrated with a 3L, 5530 series, calibration syringe (Hans Rudolph, Kansas City, Missouri, USA). The same metabolic system with identical calibration routines was used during the subsequent HIT sessions. The speed at 4.0 mmol·L^−1^ [La^−^] (V_@4mmol/L_) was calculated from the relationship between [La^−^] and speed using linear regression between the closest workload below and above 4.0 mmol·L^−1^.

Ten minutes after the submaximal test an incremental test was performed, starting at a 7% inclination and a speed of 11 km·h^−1^ which increased by 1 km·h^−1^ every minute until exhaustion. V̇O_2peak_ was calculated as the average of the two highest 30-s consecutive measurements, and for HR_peak_, the highest 1-s value was recorded (Polar RCX5, Polar, Kempele, Finland). MAS was defined as the speed where the horizontal line representing V̇O_2peak_ met the extrapolated linear regression representing the submaximal V̇O_2_/speed relationship (29), while maximal 1-min velocity achieved during the V̇O_2max_ test (V_max_) was defined as the mean velocity during the last minute of the V̇O_2peak_ test.

### Haematology

After arrival at the laboratory, before commencing the exercise testing, the participants drank 300 mL water and were placed in a semi-recumbent position for 15 min with a heat bag in their hand (to increase blood circulation in fingers), whereafter capillary blood was sampled from a fingertip for determination of haematocrit (HCT) using the microhaematocrit method (70 IU^.^mL^−1^ Hemato-Clad, Drummond, Scientific Company, Broomall, PA, USA), centrifuged (Heraeus PICO 17 Hematocrit Rotor, Thermo Electron LED GmbH, Osterode, Germany) for 5 min at 13,500 rpm and determination of haemoglobin concentration [(Hb); ABL800, Radiometer, Copenhagen, Denmark]. The mean value of three measurements for both HCT and (Hb) was used for subsequent total Hb_mass_ and intravascular volume calculations. All exercise tests were performed after obtaining this blood sample. Twenty minutes after finalising the last exercise test, the participants were again placed in a semi-recumbent position for 5 min before Hb_mass_ was determined using a modified version of the carbon monoxide (CO) rebreathing technique (OpCO, Detalo Performance, Detalo Health, Birkerød, Denmark), described in detail elsewhere (30). The participant breathed 100% O_2_ (AGA, Oslo, Norway) for 1 min before a blood sample was drawn from the fingertip (125 μl) and immediately analysed in triplicate for carboxyhaemoglobin (%HbCO; ABL80 FLEX CO-OX analyser, Radiometer, Copenhagen, Denmark). Subsequently, the participants rebreathed a bolus of chemically pure CO (AGA, Oslo, Norway) corresponding to 1.5 mL·kg^−1^ for 6 min. During the rebreathing period, O_2_ was administrated on a demand basis. The amount of CO not taken up by the participants during the rebreathing period was determined by automatic assessments of the volume of the system multiplied by the parts per million (Dräger Pac 5500; Drägerwerk AG, Lübeck, Germany) for CO remaining in the system after the rebreathing period. Four minutes after the termination of the rebreathing, a second blood sample was drawn from the fingertip (125 μl) and immediately analysed in triplicate for %HbCO. All blood values were entered into the device software where Hb_mass_, red blood cell volume (RBCV), total blood volume (BV) and PV were calculated.

### Training during intervention period

**Figure 1:**
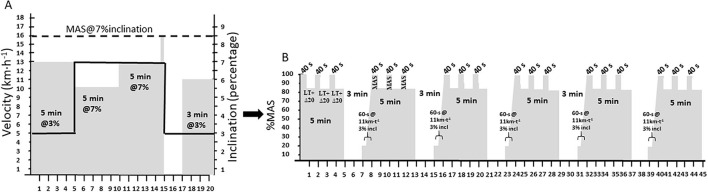
**(A)** The standardised warm-up procedure was based on a gradual change in velocity (grey area) and inclination (solid black line) that was performed prior to all high-intensity interval (HIT) sessions. **(B)** The HIT sessions consisted of 6 x 5-min varied-intensity work intervals. Each 5-min work interval started with 40 s at maximal aerobic speed (MAS) followed by 60 s at the speed equal to lactate threshold (LT) + 20% of the difference between LT and MAS. This 100-s module was then repeated three times in each 5-min work interval.

The 6-day HIT block started with one daily HIT session during the first three consecutive days, followed by one recovery day, and finally 2 days in a row with one daily HIT session. All HIT sessions in BLOCK were similar and performed as indoor roller ski skating on a large treadmill. The details of the standardised 20-min warm-up are presented in Figure 1A. The HIT sessions were performed as 6 × 5-min work intervals with varied intensity within the work intervals as described in detail in Rønnestad et al. (27) (Figure 1), which is previously observed to induce longer time ≥90% V̇O_2max_ than the same mean work interval intensity when evenly distributed (i.e., no variation in intensity) in well-trained XC skiers (27). Briefly, the recovery period between each work interval was 3 min where the first 2 min was passive in order to measure [La^−^] and give the athlete the possibility to hydrate, and the last minute before the next work interval was active (11 km·h^−1^ and 3% inclination). During the final 5 s of each recovery period, the velocity of the treadmill was increased up to the given work interval velocity. Each 5-min work interval consisted of 3 × 40 s surges at 100% of MAS, interspersed with three 1-min periods with a velocity equal to V_@4mmol/L_+20% of the difference between V_@4mmol/L_ and MAS (Figure 1). During the two first and the last HIT sessions in the block, V̇O_2_, HR, [La^−^], and rate of perceived exertion [RPE, 6–20 scale; Borg (31)] were measured. HR and V̇O_2_ were recorded at 20 s intervals during the work intervals. Time ≥90% V̇O_2peak_ and ≥90% HR_peak_ were determined as the sum of V̇O_2_ and HR Values that were ≥90% of the V̇O_2peak_ and HR_peak_, respectively, during the work intervals.

After the HIT block, a 5-day recovery period was performed, where the 1st day consisted of complete rest and 2nd day contained 30–50 min easy exercise (running or roller skiing). On the 3rd day, the XC skiers performed 15-min warm-up followed by 4 × 5-min roller skiing at moderate intensity. The 4th day consisted of 30–60 min easy running or cycling, while the 5th day contained 30-min low-intensity roller ski skating followed by 3 × 1 min with gradually increasing intensity towards MAS. Posttesting was performed on the 6th day. The CON group continued their normal training with no restrictions except for the 2 days prior to pre- and posttesting being similar to BLOCK to ensure similar recovery status during testing. During the intervention period, BLOCK had a larger amount of HIT and a lower amount of LIT and strength training compared to CON, while there was no group difference for amount of MIT (Table 1). CON performed 2.4 ± 1.3 HIT sessions while BLOCK performed 5 ± 0 HIT sessions during the experimental period. When total training load was calculated as time spent in work intensity LIT, MIT, and HIT multiplied by a factor of 1, 2, and 3, respectively [as proposed by Lucia et al. (32)], there were no differences between BLOCK and CON during the intervention (Table 1).

**Table 1:** Training data for the HIT block group (BLOCK) and the control group (CON) during the intervention period.

	**BLOCK**	**CON**	***p*-value**
LIT (60–82% HR_max_)	9:53 ± 1:54	17:29 ± 3:19	< 0.001
MIT (83–87% HR_max_)	1:09 ± 0:38	1:11 ± 0:35	0.920
HIT (88–100% HR_max_)	3:09 ± 0:42	1:39 ± 1:02	0.001
TRIMP score	1300 ± 190	1622 ± 782	0.221
Resistance training	0:09 ± 0:14	1:29 ± 1:37	0.019
Total training	14:21 ± 2:08	21:48 ± 4:28	< 0.001

Values (hours:minutes) are presented as means ± SD. LIT, low-intensity training [60–82% of maximal heart rate (HR_max_)]; MIT, moderate-intensity training (83–87% of HR_max_); HIT, high-intensity training (88–100% of HR_max_).

### Statistics

Descriptive statistics are presented as means with standard deviations (SD). For training data variables presented in Table 1, independent samples *t*-tests were used to compare values between groups (BLOCK vs. CON). To compare training responses between groups, differences in post-intervention values were modelled in linear models (ANCOVA) with pre-intervention values as the covariate and the grouping variable as the independent variable of interest. To evaluate changes in variables sampled during the training sessions, repeated measures ANOVAs were used. When significant main effects were observed, *post-hoc* tests (with Bonferroni correction) were used to identify which session(s) differed from each other. To describe the relationship between average time ≥90%V̇O_2max_ per interval training session and training responses, simple linear regression analyses were performed between the main physiological determinants of endurance performance and time ≥90% of V̇O_2max_. Interval training session reliability for oxygen consumption, heart rate, time ≥90%V̇O_2max_ and time ≥90%HR_max_ across interval sessions (interval sessions 1, 2, and 5) was determined using the intraclass correlation coefficient (ICC) and their 95% confidence intervals (CI) (two-way random model). The single rater value was reported. ICC values < 0.5 are indicative of poor reliability, values between 0.5 and 0.75 indicate moderate reliability, values between 0.75 and 0.90 indicate good reliability, and values >0.90 indicate excellent reliability (33). Conclusions regarding differences between treatments were drawn using *p-*values < 0.05 as a cutoff to declare statistical significance. All statistical analyses, except ICC analyses, were conducted in Jamovi (34). Analyses of ICC were performed using SPSS^®^ for Windows software (release 27.0.1.0; SPSS Inc., Chicago, IL, USA).

## Results

### Effects of a HIT block on maximal and submaximal velocity and oxygen consumption

**Figure 2:**
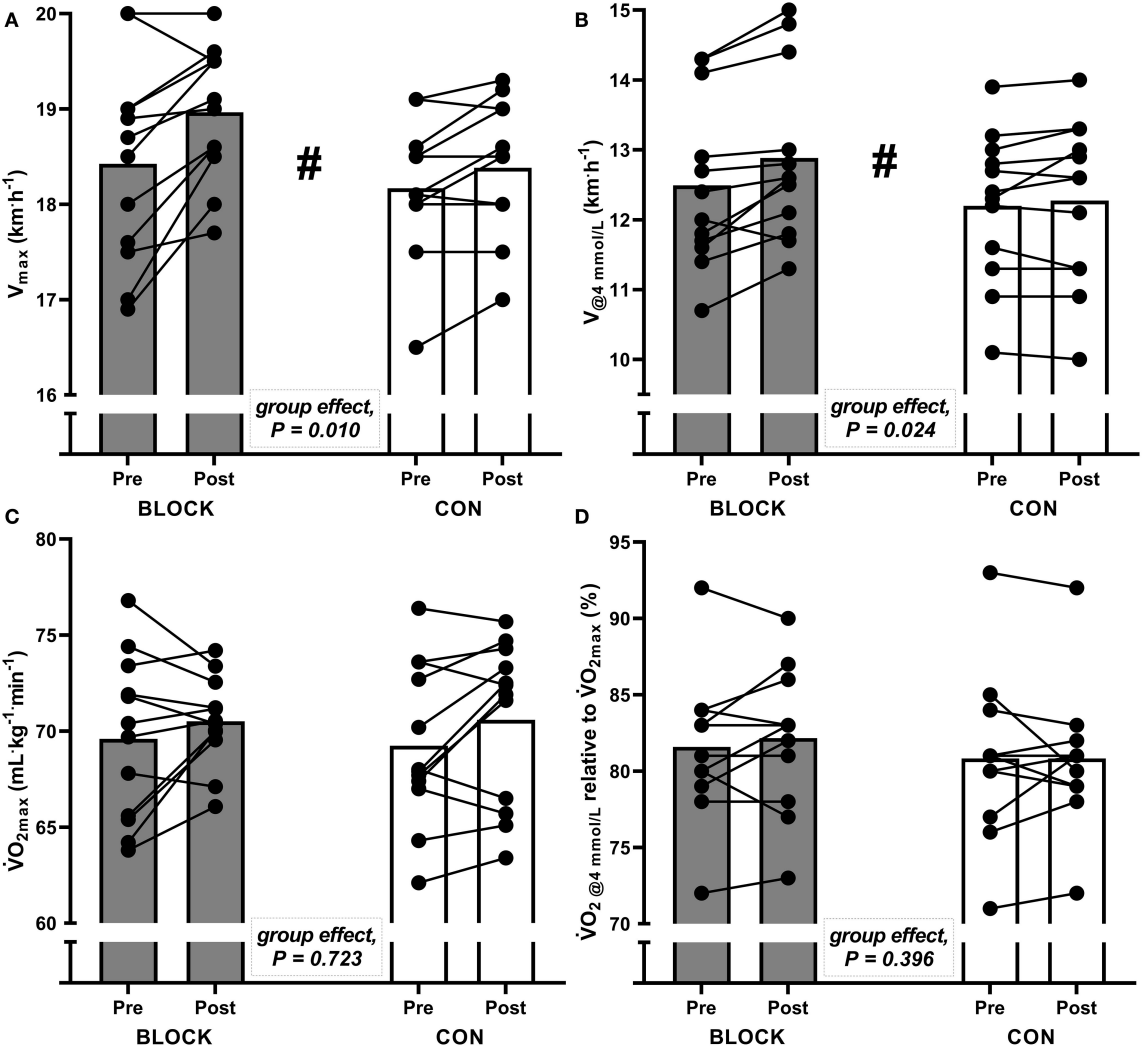
Individual (dots) and group-average (bars) values for **(A)** maximal 1-min velocity achieved during the incremental test (V_max_), **(B)** velocity corresponding to 4 mmol^.^L^−1^ blood lactate concentration (V_@4mmol/L_), **(C)** maximal oxygen uptake (V̇O_2max_), and **(D)** fractional utilisation of V̇O_2max_ at V_@4mmol/L_ (V̇O_2@4mmol/L_) for the HIT block group (BLOCK) and the control group (CON) before (pre) and after (post) the intervention. ^#^Significant different responses between BLOCK and CON.

BLOCK led to a greater improvement in V_max_ (*F*_1, 21_ = 8.0, *p* = 0.010) and V_@4mmol/L_ (*F*_1, 21_ = 6.0, *p* = 0.024) when compared to CON, although changes in V̇O_2max_ and fractional utilisation of V̇O_2max_ at V_@4mmol/L_ did not differ between groups (Figure 2).

### Effects of a HIT block on submaximal efforts

**Figure 3:**
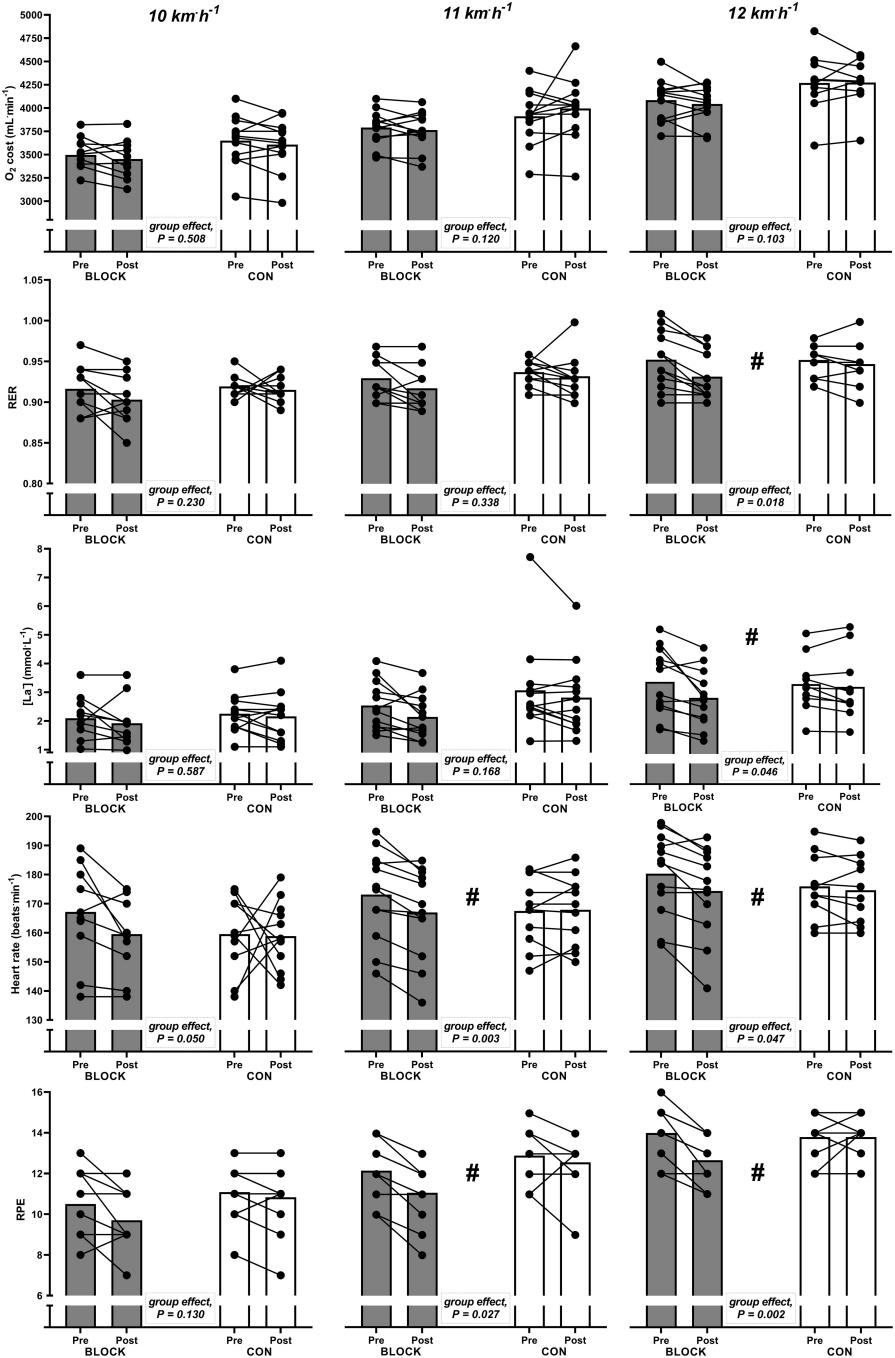
Individual (dots) and group-average (bars) values for oxygen cost (O_2_ cost), respiratory exchange ratio (RER), blood lactate concentration ([La^−^]), heart rate, and rate of perceived exertion (Borg RPE, 6–20) responses during three different submaximal efforts [10 **(left panel)**, 11 **(middle panel)**, and 12 **(right panel)** km^.^h^−1^] for the HIT block group (BLOCK) and the control group (CON) before (pre) and after (post) the intervention. ^#^Significant different responses between BLOCK and CON.

For skiing at a submaximal velocity of 12 km^.^h^−1^, BLOCK led to decreased respiratory exchange ratio (RER) compared to CON (*P* = 0.018; Figure 3). BLOCK also led to reduced [La^−^] levels at 12 km^.^h^−1^, reduced heart rate at all three velocities (10, 11, and 12 km^.^h^−1^) and reduced RPE at 11 and 12 km^.^h^−1^ compared to TRAD (Figure 3). Of note, there was a tendency towards reduced energy expenditure in BLOCK compared to CON when skiing at 12 km^.^h^−1^ (BLOCK pre, 21.1 ± 1.1 kcal^.^min^−1^; BLOCK post, 20.8 ± 1.0 kcal^.^min^−1^; CON pre, 22.0 ± 1.7 kcal^.^min^−1^; CON post, 22.0 ± 1.3 kcal^.^min^−1^; *p* = 0.073).

### Effects of a HIT block on haematological variables

For all haematological variables, training responses were similar between BLOCK and CON (Table 2).

**Table 2:** Haematological variables before (pre) and after (post) the intervention for the HIT block group (BLOCK) and the control group (CON).

**Haematological variable**	**BLOCK**		**CON**		**ΔBLOCK vs. ΔCON (*p*-value)**
	**Pre**	**Post**	**Pre**	**Post**	
Haematocrit (%)	44.9 ± 2.55	44.3 ± 1.93	45.8 ± 2.29	45.4 ± 2.57	0.469
Blood volume (mL)	6426 ± 733	6469 ± 838	6558 ± 526	6427 ± 454	0.308
Plasma volume (mL)	3527 ± 449	3572 ± 464	3632 ± 361	3509 ± 323	0.248
Haemoglobin mass (g)	1007 ± 103	1007 ± 103	1031 ± 100	1009 ± 91	0.053
Haemoglobin mass (g^.^kg body mass)	13.3 ± 0.9	13.4 ± 0.8	13.6 ± 0.7	13.5 ± 0.6	0.067

Δ, change from pre- to post-intervention.

### Exercise training variables

For an overview of data collected during HIT session numbers 1, 2, and 5, see Table 3. Mean velocity during intervals was similar across interval training sessions (*p* = 0.275; Table 3). The three interval sessions had an ICC of 0.875 (95% CI, 0.708–0.958), 0.907 (95% CI, 0.776–0.969), 0.567 (95% CI, 0.223–0.831), and 0.235 (95% CI, −0.101–0.631) for V̇O_2_, HR, time ≥90% of V̇O_2max_, and time ≥90% of HR_max_, respectively.

**Table 3:** Data collected during session 1, 2, and 5 for BLOCK.

**Interval working period (1–6)**	**Velocity (km^.^h^−1^)**	**V̇O_2_ (mL^.^min^−1^)**	**% of V̇O_2max_**	**Time ≥90% of V̇O_2max_ (mm:ss)**	**HR (beats^.^min^−1^)**	**% of HR_max_**	**Time ≥90% HR_max_ (mm:ss)**	**[La^−^] (mmol^.^L^−1^)**	**% of [La^−^] _max_**	**Borg RPE (6–20)**
**Session 1**										
Working period 1 (*n* = 12)	14.1 ± 1.0	4516 ± 456	87 ± 4	01:40 ± 01:31	184 ± 10	90 ± 4	03:13 ± 01:46	6.5 ± 0.9	50 ± 8	15.4 ± 1.4
Working period 2 (*n* = 12)	14.1 ± 1.0	4575 ± 352	87 ± 3	02:10 ± 01:30	187 ± 10	91 ± 3	03:52 ± 01:05	7.4 ± 1.1	57 ± 9	16.3 ± 1.0
Working period 3 (*n* = 12)	14.1 ± 1.0	4592 ± 390	88 ± 3	02:08 ± 01:27	189 ± 10	93 ± 2	04:17 ± 00:35	8.3 ± 1.4	64 ± 11	17.0 ± 1.0
Working period 4 (*n* = 12)	14.0 ± 1.1	4560 ± 390	87 ± 3	01:53 ± 01:26	188 ± 8	92 ± 2	04:08 ± 00:20	8.7 ± 2.0	67 ± 15	17.4 ± 0.8
Working period 5 (*n* = 12)	13.9 ± 1.1	4521 ± 340	86 ± 3	01:53 ± 01:31	188 ± 8	92 ± 2	04:00 ± 00:35	8.7 ± 2.0	66 ± 14	17.7 ± 0.8
Working period 6 (*n* = 11)	13.9 ± 1.2	4619 ± 410	88 ± 3	02:20 ± 01:16	187 ± 9	93 ± 2	04:11 ± 00:26	8.3 ± 2.0	63 ± 13	17.4 ± 0.8
*Average for session 1*	14.0 ± 1.0	4559 ± 380	87 ± 3	11:53 ± 07:57 (total time)	187 ± 9	92 ± 2	23:20 ± 04:07 (total time)	8.0 ± 1.4	60 ± 9	16.9 ± 0.7
**Session 2**										
Working period 1 (*n* = 12)	14.1 ± 0.9	4502 ± 418	86 ± 4	01:30 ± 01:28	181 ± 10	89 ± 3	02:28 ± 01:42	6.1 ± 1.1	47 ± 9	15.7 ± 1.2
Working period 2 (*n* = 12)	14.1 ± 0.9	4607 ± 359	88 ± 3	02:33 ± 01:19	185 ± 10	91 ± 3	03:47 ± 00:52	7.1 ± 1.7	55 ± 14	16.5 ± 1.2
Working period 3 (*n* = 12)	14.1 ± 0.9	4621 ± 380	88 ± 3	02:40 ± 01:22	186 ± 10	92 ± 2	03:57 ± 00:37	7.8 ± 1.7	60 ± 13	17.3 ± 1.0
Working period 4 (*n* = 12)	14.0 ± 0.8	4622 ± 378	88 ± 3	02:30 ± 01:28	186 ± 10	91 ± 3	03:50 ± 00:41	7.9 ± 1.9	61 ± 14	17.4 ± 1.0
Working period 5 (*n* = 12)	13.9 ± 0.9	4611 ± 411	88 ± 4	02:25 ± 01:36	187 ± 10	92 ± 3	03:53 ± 01:01	7.8 ± 1.9	60 ± 14	17.5 ± 0.9
Working period 6 (*n* = 11)	13.9 ± 0.8	4597 ± 389	88 ± 3	02:33 ± 01:07	189 ± 9	93 ± 2	04:15 ± 00:18	7.5 ± 1.8	57 ± 10	17.3 ± 1.2
*Average for session 2*	14.0 ± 0.9	4634 ± 381	88 ± 3	13:58 ± 06:54 (total time)	186 ± 10^£^	91 ± 2	21:48 ± 04:44 (total time)	7.4 ± 1.6	55 ± 10	17.0 ± 0.9
**Session 5**										
Working period 1 (*n* = 12)	14.1 ± 0.9	4615 ± 353	88 ± 3	02:17 ± 01:16	177 ± 10	88 ± 5	01:50 ± 01:59	5.9 ± 1.1	46 ± 8	14.9 ± 1.3
Working period 2 (*n* = 12)	14.2 ± 0.9	4678 ± 338	89 ± 4	02:52 ± 01:15	182 ± 9	89 ± 2	02:58 ± 01:15	6.6 ± 1.3	50 ± 8	16.1 ± 0.9
Working period 3 (*n* = 12)	14.2 ± 0.9	4682 ± 370	89 ± 3	02:58 ± 00:52	183 ± 9	90 ± 2	03:08 ± 01:16	7.2 ± 1.6	55 ± 11	16.4 ± 0.9
Working period 4 (*n* = 12)	14.1 ± 0.8	4679 ± 384	89 ± 4	02:35 ± 01:27	183 ± 9	90 ± 2	03:18 ± 01:10	7.2 ± 1.5	55 ± 9	16.5 ± 1.0
Working period 5 (*n* = 12)	14.0 ± 0.8	4632 ± 373	89 ± 4	02:27 ± 01:25	183 ± 9	90 ± 2	03:22 ± 01:13	6.8 ± 1.8	52 ± 11	16.5 ± 1.0
Working period 6 (*n* = 12)	14.1 ± 0.9	4619 ± 307	88 ± 5	02:40 ± 01:33	185 ± 8	91 ± 1	03:25 ± 01:09	7.3 ± 1.8	56 ± 11	16.7 ± 1.4
*Average for session 5*	14.1 ± 0.9	4651 ± 342	89 ± 3^£^	15:48 ± 06:37 (total time)	182 ± 9^£, §^	90 ± 2^£^	18:02 ± 06:57 (total time)	6.8 ± 1.4^£^	52 ± 8	16.2 ± 0.9^£, §^
*Session effect (P-value)*	0.275	0.210	0.030	0.151	0.001	0.003	0.033	0.060	0.045	0.006

All interval sessions were conducted at 7% inclination on the same treadmill. For calculation of % of V̇O_2max_ and [La^−^]_max_, baseline values were used. V̇O_2_, oxygen uptake per time unit; HR, heart rate; [La^−^], blood lactate concentration; RPE, rate of perceived exertion on a scale from 6–20.

^£^Statistically different from session 1.

^§^Statistically different from session 2. Alpha level at *p* < 0.05.

For BLOCK, there was a tendency towards a positive relationship between time ≥90% of V̇O_2max_ and improvements in V̇O_2max_ in both absolute values and expressed per kilo body mass, while there was no such relationship for the other main training outcomes (Table 4).

**Table 4:** Simple linear regression analyses for the relationship between time ≥90% of V̇O_2max_ during HIT sessions and main training responses in BLOCK.

**Analysis**	***n***	**Intercept (95% CI)**	**Slope (95% CI)**	***r***	***p*-value**
**Time (avg sec**^.^**session**^−1^**)** ≥**90% V̇O**_2max_ **vs**.
ΔV̇O_2max_ (mL^.^kg^−1.^min^−1^)	12	769 (551, 988)	70 (−7, 147)	0.538	0.071
ΔV̇O_2max_ (mL^.^min^−1^)	12	787 (570-1003)	0.87 (−0.14, 1.88)	0.518	0.084
ΔV_max_ (km^.^h^−1^)	12	731 (395, 1066)	189 (−254, 632)	0.288	0.363
ΔV_@4mmol/L_ (km^.^h^−1^)	12	920 (549, 1292)	−222 (−948, 504)	0.211	0.511

*r*, Pearson's *r*; Δ, post-value – prevalue; V̇O_2max_, maximal oxygen uptake per time unit; V_max_, maximal velocity achieved during the incremental test; V_@4mmol/L_, velocity corresponding to 4 mmol^.^L^−1^blood [La^−^].

## Discussion

The primary findings of the present study support our initial hypothesis, which means that BLOCK induces a superior improvement in the endurance performance indicators V_max_ and velocity_@4mmol/L_ compared to CON. This might be related to the observed larger reduction in RER, [La^−^], HR, RPE, and the tendency towards less energy expenditure during submaximal exercise. Furthermore, the results indicate that the reliability of accumulated time ≥90% V̇O_2max_ between exercise sessions during a HIT block is moderate, implying that there is a certain session-to-session variation for the individual athlete for this variable.

The BLOCK training, consisting of 5 HIT sessions for 6 days followed by 5-day recovery, improved the indicator of performance, V_max_, by 3%, displaying a large effect of BLOCK compared to CON. Although the present study did not measure endurance performance directly, it has been observed that V_max_ distinguishes the endurance performance in well-trained skiers, cyclists, and long-distance runners and predicts endurance performance (35–39). The reason for this is probably that V_max_ is influenced not only by V̇O_2max_ and work economy, but also incorporates anaerobic capacity and neuromuscular characteristics (39). The present finding of superior improvements in V_max_ in BLOCK agrees with most previous studies investigating the effect of a HIT block (6, 8–10). Similarly, the secondary endurance performance indicator in this study, V_@4mmol_, had a larger increase in BLOCK than CON, which also agrees with other studies measuring the effect of a HIT block on measures of lactate threshold velocity or lactate threshold power output reporting either significant (7–10) or numerical advantages (6, 11). In accordance with the present study, case studies of world-class endurance athletes report improved threshold velocity without any changes in V̇O_2max_ (40–42). Interestingly, it has been observed that the HIT session with the longest time at V̇O_2max_ (measured during the first and last HIT sessions) induced the largest improvement in threshold power after 4 weeks of training (43). However, the latter study is amongst the few that actually have observed that longer time ≥90%V̇O_2max_ is associated with superior training adaptations and there was no correlation between time ≥90%V̇O_2max_ and training adaptations (43). Most studies focusing on time at high intensity in terms of V̇O_2_ are acute studies just comparing different HIT sessions without any measures of training adaptations. Accordingly, there is a lack of knowledge of the actual importance of time at high percentages of V̇O_2max_ for endurance training adaptations. In the present HIT microcycle, there was a substantial time ≥90% V̇O_2max_, as measured during HIT session numbers 1, 2, and 5, which might be linked to the superiority of BLOCK. Furthermore, within BLOCK there was a tendency towards that time ≥90%V̇O_2max_ during the HIT sessions could estimate the improvement in V̇O_2max_ (*r* = 0.54, *P* = 0.071), indicating a role for time ≥90% V̇O_2max_ for training adaptations. That being said, a correlation does not establish the cause of an effect, and BLOCK did not achieve a larger improvement in V̇O_2max_ than CON. Furthermore, time ≥90% V̇O_2max_ was not able to explain changes in V_max_ or V_@4mmol_.

The seemingly performance-enhancing effect of a HIT microcycle on XC skiers in the present study is in agreement with a case report on the Norwegian female national team in XC skiing (19). Karlsen et al. (19) observed that concomitant with the introduction of systematic HIT block periodization there was a clear improvement in the International Ski Federation's ranking points, indicating improved performance. On the contrary, a study on well-trained junior XC skiers did not observe any difference in effects between a HIT block approach compared to an evenly distributed HIT approach (12). However, as the authors discuss, no advantage of HIT blocking could be related to performing a large amount of LIT the last week prior to posttesting, a long break between every 4-min HIT work interval (6 min), and a large HIT stimulus (nine sessions in 1 week) with no specific recovery period before posttesting. The importance of reducing the training load both before and after a HIT microcycle has recently been emphasised (19, 44) and implemented in the present study design.

The main physiological determinants of endurance performance are V̇O_2max_, fractional utilisation of V̇O_2max_ and work economy (45). Theoretically, one or more of these determinants should explain the superior adaptation on endurance performance indicators of BLOCK. One of the main arguments of using HIT microcycles is to improve V̇O_2max_ (19) and that is also the main argument for designing intervals that maximises time at high V̇O_2_, like ≥90% V̇O_2max_ (1, 20, 46). However, in the present study there was no difference between BLOCK and CON in either V̇O_2max_ or blood variables such as Hb_mass_, which is greatly related to V̇O_2max_ (13). No significant change in V̇O_2max_ is in agreement with some HIT microcycle studies (6, 8, 10, 12), but in contrast to others who observe increased V̇O_2max_ after a HIT microcycle (7, 9, 11). In a similar cohort as the present, i.e., elite XC skiers, no change in V̇O_2max_ during the training year has been observed (17), which was explained by the high all-year around training volume that includes both HIT and LIT. Furthermore, in well-trained endurance runners there are observations that short-term HIT does not seem to improve V̇O_2max_ (47–49), and a recent meta-analysis indicates that HIT interventions have little effect on changes in V̇O_2max_ in well-trained runners (44). The latter findings emphasise the difficulties in improving V̇O_2max_ during a short, 2-week period in well-trained XC skiers and that other physiological determinants of endurance performance most likely should account for the superior improvement in endurance indicators in BLOCK in the current study. Accordingly, multiple studies in well-trained endurance athletes report improvements in endurance performance indices without being able to relate it to changes in V̇O_2max_ (6, 10, 50, 51).

There were no changes in fractional utilisation of V̇O_2max_ [measured as V̇O_2_ at 4.0 mmol·L^−1^ [La^−^]] in either BLOCK or CON, which agrees with most short-term studies (10, 11, 52). However, BLOCK seems to improve the cost of submaximal exercise, as evident by a significant lowering of RER, [La^−^], HR, RPE, and tendency towards less energy expenditure at submaximal exercise (12 km·h^−1^) compared to CON. The tendency towards reduced submaximal energy expenditure is probably the main contributor to superior improvement in V_max_ and V_@4mmol_ in BLOCK. In general, work economy is thought to be relatively stable, and for instance in elite cyclists, no changes in work economy during a 6-month period has been observed (53, 54). In contrast to cycling, XC skiing is a highly technically demanding exercise with involvement of both arms and legs which increases the potential for technical improvements to affect the energy cost (17) and could explain some of the changes found in the present study. Also, other studies on well-trained XC skiers have observed improved work economy within a relatively short time frame (55, 56). It can be speculated that the larger amount of roller ski skating at HIT intensity induced a larger improvement in technical abilities in BLOCK than CON and that this contributes to the improvements in submaximal physiological and perceptual effort. However, CON had a larger amount of training at LIT intensity than BLOCK which can be argued to have a larger specificity towards the submaximal exercise intensities where the superior adaptations occurred in BLOCK. Of note, all XC skiers were used to roller ski testing on treadmill, and all of them performed a familiarisation test prior to pretest and thereby minimised the potential learning effect. Thus, it can be suggested that it was the physiological stimulus (like time ≥90% V̇O_2max_) that induced adaptations responsible for superior improvement in V_max_ and velocity@4mmol·L^−1^ in BLOCK, and not changes in technical abilities *per se*. The latter is supported by the observations that the HIT sessions that induced the longest time at V̇O_2max_ induced the largest improvement in lactate threshold power output after 4 weeks of training (43). Evidently, the present HIT microcycle induced a substantial time ≥90% V̇O_2max_, as measured during HIT session numbers 1, 2, and 5, which might contribute to explain the superiority of the HIT microcycle. The present study is not designed to investigate possible mechanistic explanations for our observations, but speculatively it is observed that cellular stress occurs in proportion to the training intensity (57, 58), and it has been suggested that the stimulus for adaptation is indeed exercise intensity-dependent up to V̇O_2max_ (46). It can therefore be hypothesised that BLOCK induced peripheral adaptations associated with improved V_@4mmol_ like capillarization, increased size, function, and number of mitochondria, and increased activity of enzymes involved in aerobic respiration (59). It should be mentioned that in study designs like this, we cannot exclude a placebo effect, although this probably is more unlikely in a group of well-trained athletes. Participants in BLOCK knew they were performing a HIT microcycle, and speculatively, this may have motivated them to push harder in the incremental test compared to CON.

During the present HIT microcycle, the mean HR and [La^−^] during the work intervals were ~91% of HR_max_ and 7–8 mmol·L^−1^, respectively. This is similar to the values as observed in a case report of the Norwegian female national team in XC skiing describing their block periodization of HIT (19). A trend towards progressive increase in HR and [La^−^] from the first work interval to the last interval was also evident in both studies. There seems to be growing evidence for the importance of time ≥90% of V̇O_2max_ to maximally stress cardiorespiratory factors in well-trained endurance athletes (20–22). The present study adds knowledge on V̇O_2_ responses during a HIT microcycle, where there was a gradual increase in mean percentage of V̇O_2_ during the work intervals from the first to the last HIT session. A similar numerical tendency from the first to the last HIT session was observed in time ≥90% V̇O_2max_ (from ~12 to ~16 min), indicating a strong physiological stimulus throughout the HIT block. Interestingly, time ≥90% V̇O_2max_, mean percentage of HR_max_, mean [La^−^], and RPE is within the same range as observed acutely during one 5 × 5-min roller ski double-poling HIT session with similar work intervals as in the present study (27). The latter agrees with the findings of similar physiological stimulus across different exercise modes in XC skiing (19). However, despite mean values of time ≥90% V̇O_2max_ are in the same range within the sessions in the present HIT block and these values also are in agreement with a HIT session while double poling on roller skis, does not necessarily say anything about the reliability of time ≥90% V̇O_2max_ between HIT sessions.

The ICC of time ≥90% V̇O_2max_ in the present study was 0.57, which indicates moderate reliability (33). This is a lower reliability than the ICC of 0.80 observed on time ≥90% V̇O_2max_ during a repeated intermittent treadmill running protocol in distance runners (25). The lower reliability in the present study compared to the Midgley et al. (25) study can be related to the longer total exercise duration (30 min vs. 14–15 min, respectively), longer time ≥90% V̇O_2max_ (12–15 min vs. 8 min, respectively), different HIT protocols, different movement pattern (XC skiing vs. running, respectively) and potentially induced fatigue during the HIT block. Furthermore, the 95% confidence interval in the present study indicates a large variation where the reliability ranges from poor (ICC = 0.22) to good (ICC = 0.83). In practicality, this reliability for time ≥90% V̇O_2max_ demands a careful interpretation of a single HIT session in XC skiing, and multiple sessions are therefore recommended to establish a good picture for time ≥90% V̇O_2max_ for the individual athlete. Of note, HR measurements are often used as a surrogate measurement for V̇O_2_, but in the present study, the reliability of time ≥90% HR_max_ is poor (ICC = 0.235; 95% CI, −0.101 to 0.631), indicating that HR measurements should be used carefully.

Mean HR, [La^−^], and RPE from the work intervals were reduced from the first to the fifth HIT session in BLOCK, with an increase in mean percentage of V̇O_2_ during the work intervals in the same period. Reduced HR and [La^−^] can be indices of both positive training adaptations and, to the contrary, overreaching (60). However, since perceived effort, measured as RPE, was also reduced, it might be suggested that these data indicate a positive training adaptation in BLOCK and that the HIT microcycle intervention was well-tolerated. The latter is supported by the superior improvement in V_max_ and V_@4mmol_ in BLOCK vs. CON.

## Conclusions

In well-trained XC skiers, BLOCK induced superior changes in indicators of endurance performance compared with CON, while time ≥90% of V̇O_2max_ during the HIT sessions in the 6-day block had a moderate reliability.

## Data availability statement

The raw data supporting the conclusions of this article will be made available by the authors, without undue reservation.

## Ethics statement

The study was reviewed and approved by the Local Ethical Committee of the Section for Health and Exercise Physiology, Inland Norway University of Applied Sciences. The participants provided their written informed consent to participate in this study.

## Author contributions

BR, JH, and KB planned and designed the study. KB and JH performed the data collection. KSM, KB, and BR analysed and presented the data. KSM, BR, KB, and JH authored and finalised the manuscript for publication and have approved the final manuscript. All authors contributed to the article and approved the submitted version.

## Conflict of interest

The authors declare that the research was conducted in the absence of any commercial or financial relationships that could be construed as a potential conflict of interest.

## Publisher's note

All claims expressed in this article are solely those of the authors and do not necessarily represent those of their affiliated organizations, or those of the publisher, the editors and the reviewers. Any product that may be evaluated in this article, or claim that may be made by its manufacturer, is not guaranteed or endorsed by the publisher.
